# Ratio of Intensities of Blue and Red Light at Cultivation Influences Photosynthetic Light Reactions, Respiration, Growth, and Reflectance Indices in Lettuce

**DOI:** 10.3390/biology11010060

**Published:** 2022-01-01

**Authors:** Lyubov Yudina, Ekaterina Sukhova, Maxim Mudrilov, Vladimir Nerush, Anna Pecherina, Alexandr A. Smirnov, Alexey S. Dorokhov, Narek O. Chilingaryan, Vladimir Vodeneev, Vladimir Sukhov

**Affiliations:** 1 Department of Biophysics, N.I. Lobachevsky State University of Nizhny Novgorod, 603950 Nizhny Novgorod, Russia; lyubovsurova@mail.ru (L.Y.); n.catherine@inbox.ru (E.S.); mtengri@yandex.ru (M.M.); panzerblitz@mail.ru (V.N.); pechyorinaa@gmail.com (A.P.); v.vodeneev@mail.ru (V.V.); 2 Lighting Laboratory, Federal State Budgetary Scientific Institution “Federal Scientific Agroengineering Center VIM” (FSAC VIM), 109428 Moscow, Russia; as984788@gmail.com; 3 Department of Closed Artificial Agroecosystems, Federal State Budgetary Scientific Institution “Federal Scientific Agroengineering Center VIM” (FSAC VIM), 109428 Moscow, Russia; dorokhov.vim@yandex.ru; 4 Agricultural Materials Laboratory, Federal State Budgetary Scientific Institution “Federal Scientific Agroengineering Center VIM” (FSAC VIM), 109428 Moscow, Russia; narek-s@list.ru

**Keywords:** LEDs, red light, blue light, photosynthesis, respiration, reflectance indices, growth, cultivation, lettuce

## Abstract

**Simple Summary:**

Illumination is an important factor for plant life because light is the basis of photosynthesis and productivity, the regulator of physiological processes, and a potential cause of damage. The development of LED technology has contributed to increasing the efficiency of illumination during plant cultivation through the use of light sources with narrow spectral bands. However, the characteristics of influence of light sources with different spectra on specific species of agricultural plants require further investigation. In the present work, we analyzed the influence of two variants of LED illumination (with increased intensity of red or blue light) on physiological processes in lettuce. These variants were selected because they corresponded to two maximums of photosynthetic light absorption. It was shown that, under the increased intensity of the blue light, both respiration and cyclic electron flow were stimulated; theseprocesses are known to be related to stress changes in plants. In contrast, under the increased intensity of the red light, linear electron flow was stimulated, a process that is related to plant productivity, and the biomass during cultivation was increased. The reflectance of leaves was also dependent on the variant of illumination. In the future, our results can be used to increase the efficiency of lettuce cultivation.

**Abstract:**

LED illumination can have a narrow spectral band; its intensity and time regime are regulated within a wide range. These characteristics are the potential basis for the use of a combination of LEDs for plant cultivation because light is the energy source that is used by plants as well as the regulator of photosynthesis, and the regulator of other physiological processes (e.g., plant development), and can cause plant damage under certain stress conditions. As a result, analyzing the influence of light spectra on physiological and growth characteristics during cultivation of different plant species is an important problem. In the present work, we investigated the influence of two variants of LED illumination (red light at an increased intensity, the “red” variant, and blue light at an increased intensity, the “blue” variant) on the parameters of photosynthetic dark and light reactions, respiration rate, leaf reflectance indices, and biomass, among other factors in lettuce (*Lactuca sativa* L.). The same light intensity (about 180 µmol m^−2^s^−1^) was used in both variants. It was shown that the blue illumination variant increased the dark respiration rate (35–130%) and cyclic electron flow around photosystem I (18–26% at the maximal intensity of the actinic light) in comparison to the red variant; the effects were dependent on the duration of cultivation. In contrast, the blue variant decreased the rate of the photosynthetic linear electron flow (13–26%) and various plant growth parameters, such as final biomass (about 40%). Some reflectance indices (e.g., the Zarco-Tejada and Miller Index, an index that is related to the core sizes and light-harvesting complex of photosystem I), were also strongly dependent on the illumination variant. Thus, our results show that the red illumination variant contributes a great deal to lettuce growth; in contrast, the blue variant contributes to stress changes, including the activation of cyclic electron flow around photosystem I.

## 1. Introduction

Light is a key factor that affects the lives of plants [1,2,3] and can play both positive and negative roles for these organisms. Photosynthesis is likely to be the main target of the light action because it requires light as an energy source [4,5,6,7], is regulated by the intensity, spectra, and time regime of illumination [1,2,3,8,9,10,11,12,13], and can be damaged under the high-intensity light [10,14,15,16,17]. These properties of photosynthesis can be used for the development of new methods that can improve its efficiency by regulating the parameters of illumination by LEDs [2,3]. It is known that LED illumination can have narrow spectral band; its intensity and time regime are regulated in wide range [18]. As a result, combinations of LEDs with different spectra are important for forming optimal light conditions for photosynthetic processes in specific species and cultivars of agricultural plants [2,3,18].

There are several ways that light spectra can influence photosynthetic processes. First, this influence can be caused during the light absorption process in the photosynthetic light-harvesting complexes and by further electron transfer in the photosynthetic electron transport chain. It is traditionally considered that red and blue light are mainly used by the photosynthetic machinery; however, new research has shown that green light can participate in supporting photosynthetic processes at the level of the leaf mesophyll [2,3]. The induction of the energy-dependent component during the non-photochemical quenching of chlorophyll fluorescence (NPQ) [1,10,19,20], the activation of cyclic electron transport around photosystem I (CEF) [8,21], and the migration of light-harvesting complex II from photosystem II (PSII) to photosystem I (PSI) (or “state transition”) [20,22], among other processes, can be activated in this way. Also, the light absorption process that takes places in the photosynthetic light-harvesting complexes lead to photodamage, which is related to the production of reactive oxygen species [21,22].

Second, the influence of illumination on photosynthetic processes may be related to the activity of specific photoreceptors, including phytochromes, which are sensitive to red and far-red light, as well as cryptochromes and phototropins, which are sensitive to blue light [23,24,25]. According to [26], it is known that all receptors can participate in light-induced stomata opening and, as a result of this, they are able to regulate photosynthetic processes. Additionally, there are other photosynthetic regulation mechanisms that work on basis of the activity of these receptors: phytochromes can regulate photosynthetic tolerance to photodamage [27], participate in stomata development [23], and influence the synthesis of carotenoids [28,29] and chlorophylls [30]; cryptochromes participate in the development of chloroplasts and stomata [26] and in the synthesis of carotenoids [28]; and phototropins play an important role in the light-induced movement of chloroplasts [3,31]. Additionally, the influence of the blue light-induced activation of phototropins on photosynthesis could potentially be related to the stimulation of the H^+^-ATP-ase in the plasma membrane [26,32] because changes in the activity of this ATP-ase are an important mechanism of photosynthetic regulation in plants [33,34,35,36].

Because of this, it is possible that different mechanisms influence photosynthetic processes during plant cultivation under controlled light spectra. However, the induction of photosynthetic changes is not a unique mechanism of influence that these spectra have on plants. For example, it is known that the light-induced activation of phytochromes [23] and cryptochromes [24] can modify the growth and development of plants. There are other processes that are affected by this activation, including the gravitropic responses of plants, flowering, and circadian clock regulation [23,24]; phytochromes can participate in freezing tolerance; and cryptochromes play an important role in magnetoreception and can participate in programmed cell death. It is interesting that the activation of phytochromes by red light can decrease respiration processes in the mitochondria [37]; the result shows additional ways in which illumination can influence energy production in plants as well as their productivity.

Thus, the influence of spectra of illumination on plant cultivation is the result of the interaction of numerous processes (including photosynthetic processes) that can be affected by light with different spectral bands. In general ([38,39,40,41,42]), blue light stimulates chloroplast development, the production of photosynthetic pigments, and photosynthetic processes and inhibits growth; red light has the opposite influence. Combinations of LEDs with both red and blue spectra (or with the red, blue, and white/green spectra) are an effective tool for the plant cultivation because they support relatively high photosynthetic and growth processes [38,39,40,43,44,45]; in contrast, adding far-red light can decrease the growth and photosynthetic parameters in plants [43]. It is important to note that adding even a small portion of blue light (<10–20%) to red light can strongly stimulate photosynthetic and growth processes [46,47].

However, the influence of illumination spectra on photosynthesis can be intricate, especially, when different combinations of different spectral bands are used during plant cultivation. For example, adding weak blue light during the plant cultivation strongly increases photosynthetic CO_2_ assimilation (A_hv_) and the chlorophyll content in radish but weakly influences these parameters in lettuce and spinach [46]. Increasing the portion of blue light that is influenced during plant cultivation from 0 to 60% decreases growth parameters and increases the chlorophyll content in lettuce [41]; however, using 100% blue light stimulates growth and decreases the content of these pigments. Work [40] shows that the linear electron flow (LEF) and chlorophyll a/b ratio in tomato plants are strongly increased when using a combination of red and blue light during plant cultivation in comparison to these parameters when using the blue, red, or white light; in contrast, when using blue light, the maximum content of rubisco is observed. The results from [40] additionally show the complexity of the light influence on photosynthetic processes because (i) the increase of LEF should stimulate photosynthetic CO_2_ assimilation, (ii) the decrease in the rubisco content should decrease this assimilation, and (iii) the increase of the chlorophyll a/b ratio (i.e., decrease in content of light-harvesting complex II in comparison to the content of core of PSII [48]) can also influence LEF and CEF. It is also known [38] that increasing the portion of the blue light during the cucumber cultivation stimulates dark respiration rate (R); this stimulation can influence relations between A_hv_ and plant productivity. These points show that further investigations into the influence of the spectral characteristics of the cultivation light on photosynthesis and other physiological processes in plants remains a topical task.

The aim of the present work was the analysis of influence of two variants of illumination spectra during cultivation on the physiological parameters of lettuce, including the parameters of photosynthetic dark and light reactions, dark respiration rate, growth parameters, and the main reflectance indices of the leaves. Green leaf lettuce (*Lactuca sativa* L.) was used in this investigation because it is an important agricultural plant that is widely cultivated under artificial illumination (including illumination by LEDs with narrow spectral bands [49,50,51,52]). The first variant of illumination (the “red” variant) included red and white light with a small portion of the blue light (<10% from the total light intensity and 13% from the red light intensity) because this cultivation variant causes relatively high rates of growth and photosynthesis in lettuce [41,51]. The second illumination variant included the same portion of white light, a relatively small portion of the red light, and a relatively large portion of the blue light (34% of the total light intensity and about 150% of intensity of the red light); this ratio between the intensities of the blue and red light corresponded to the strong effect of blue light that is seen in lettuce during cultivation when a combination of red and blue LED light is used [41,51].

## 2. Materials and Methods

### 2.1. Plant Materials and Light Treatment

The green leaf lettuce (*Lactuca sativa* L.) cultivar “Azart” was used in the investigation. The plants were cultivated from seeds in pots containing a cube of mineral wool (1 plant per pot); 15 pots were placed on each pallet. Medium Flora Series^®^ (GHE, Fleurance, France) was used for cultivation. Seeds were germinated for 3 days without illumination; after that the lettuce plants began to receive light treatment through the use of an LED system. The light treatment was not varied at all during the plant cultivation process.

**Figure 1:**
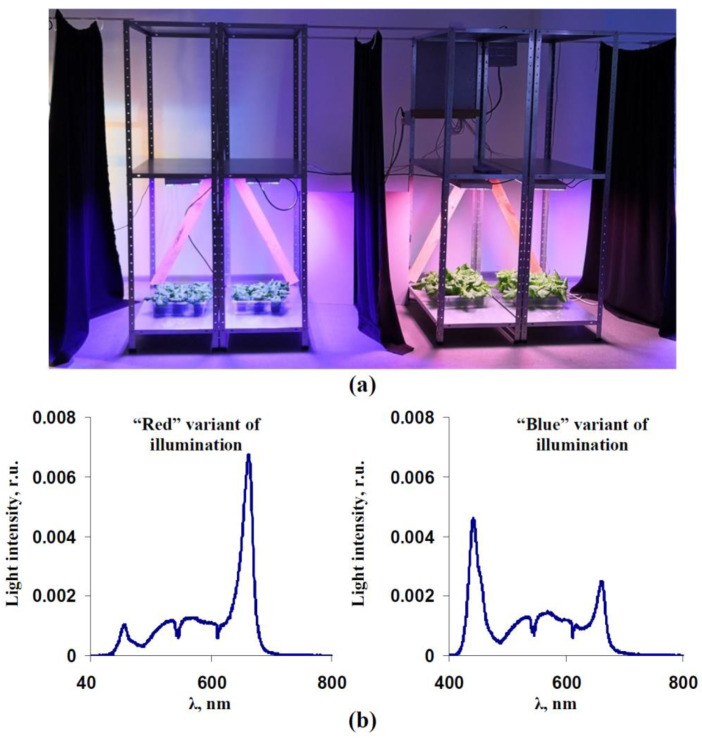
(**a**) Photo of the lettuce cultivation system. The system controlled the light intensity of three types of light-emitting diodes (LEDs, including blue, red, and white light sources) on each shelf that was used for plant cultivation. (**b**) Illumination spectra that were used during lettuce cultivation. The spectra were normalized on the total sum of intensities within 400–800 nm. The high intensity of the red LEDs and low intensity of the blue LEDs were used in the “red” illumination variant. The high intensity of the blue LEDs and low intensity of the red LEDs were used in the “blue” illumination variant. The intensity of the white LEDs was constant for both variants. The total light intensity was about 180 µmol m^−2^s^−1^. Light peaks were observed at 445 nm, about 570 nm, and 660 nm (the blue variant) and at 452 nm, about 570 nm, and 660 nm (the red variant).

A plant illumination system developed earlier (see [53] for details) was used as a light source. Briefly, the system included four types of LEDs (4000 K white LEDs, blue LEDs with maximums at 440–460 nm, red LEDs with maximums at 630–660 nm, and far-red LEDs with maximums at 730–740 nm, VANQ technology Co., Ltd., Shenzhen, China), which were integrated into the LED units. In each unit, different types of LEDs were placed line by line on the aluminum base; a common water-cooling system was integrated into this base. The maximal power of the single unit was 300 W. The intensity of each type of LED in the unit was independently regulated from 0 to 100% using pulse-width modulation (500 Hz) on basis of the power source IPC60-700TU (TC “Argos-Trade”, Saint Petersburg, Russia). A photo of these systems with the lettuce plants is shown in Figure 1a.

In our investigation, we only used red, blue, and white LEDs (intensities of far-red LEDs were zero). Two illumination variants were used: (i) red light with an increased intensity (“red” variant), which included 53% red light and 7% blue light (% were calculated from the total light intensity) and blue light with an increased intensity (“blue” variant), which included 23% red light and 34% blue light (% were calculated from the total light intensity). Intensity of the light from the white LEDs was the same for both variants; these broad-band LEDs were mainly used to provide light that was in the green and yellow spectral range (about 40% from the total light intensity).

The spectra of light in the red and blue variants were measured using a FLAME-S-VIS-NIR spectrometer (Ocean Optics, Dunedin, FL, USA). These spectra included three maximums (Figure 1b): 445 nm, about 570 nm, and 660 nm (the blue variant) and 452 nm, about 570 nm, and 660 nm (the red variant). The intensities of the both illumination variants were about 180 µmol m^−2^s^−1^; a Thorlabs PM100D optical power meter (Thorlabs Inc., Newton, MA, USA) with an S120VC sensor (200–1100 nm) was used to control the light intensity.

The light/dark intervals were 16/8 h. The air temperature and humidity in the vegetation room were about 23 °C and 50%. The plants were periodically irrigated through the used medium; the content of this medium that was in the cube of mineral wool (pot) was more than 60% from the maximal medium content. The lettuce plants were cultivated for 32 days; after that, the experiment was terminated.

### 2.2. Measurements of Reflectance Indices in Lettuce Leaves

Leaf reflectance was measured using a handheld PolyPen RP 410 UVIS systems (Photon Systems Instruments, Drásov, Czech). Leaf reflectance measurements were performed after 18, 25, and 32 days of cultivation. The reflectance spectra of 10 plants were measured in each illumination variant and at each cultivation duration period. Three spectral measurements were performed in different leaves of each individual plant.

PolyPen RP 410 UVIS software automatically calculated the main reflectance indices [54] on the basis of the previously measured reflectance spectra. We used the Normalized Difference Vegetation Index (NDVI) [55], Simple Ratio Index (SR) [55], Optimized Soil-Adjusted Vegetation Index (OSAVI) [56], Simple Ratio 554/677 Greenness Index (G) [57], Modified Chlorophyll Absorption in Reflectance Index (MCARI) [57,58], Modified Chlorophyll Absorption in Reflectance Index 1 (MCARI1) [57,58], Transformed Chlorophyll Absorption Ratio Index (TCARI) [58], Triangular Vegetation Index (TVI) [57,58], Zarco-Tejada and Miller Index (ZMI) [57], Simple Ratio Pigment Index (SRPI) [59], Normalized Phaeophytinization Index (NPQI) [57], Photochemical Reflectance Index (PRI) [60,61,62], Normalized Pigment Chlorophyll Index (NPCI) [63], Carter Indices 1 and 2 (Ctr1 and Ctr2) [57], Lichtenthaler Indices 1 and 2 (Lic1 and Lic2) [57], Structure Intensive Pigment Index (SIPI) [64], Gitelson and Merzlyak Indices 1 and 2 (GM1 and GM2) [65], Anthocyanin Reflectance Indices 1 and 2 (ARI1 and ARI2) [66], Carotenoid Reflectance Indices 1 and 2 (CRI1 and CRI2) [67], and Ratio Difference Vegetation Index (RDVI) [57]. The spectral bands that were used for the calculation of these indices as well as the equations that were used for those calculations are shown in Table S1.

### 2.3. Measurements of Parameters of Photosynthesis, Respiration, and Transpiration in Lettuce Leaves

The parameters for photosynthesis, respiration, and transpiration were measured using a standard system (Heinz Walz GmbH, Effeltrich, Germany) that included the gas analyzer GFS-3000, PAM-fluorometer Dual-PAM-100, and common measuring head Dual-PAM gas-exchange Cuvette 3010-Dual. The measurements of these parameters in the lettuce leaves were performed after 18, 25, and 32 days of plant cultivation. A total of 5–7 measurements were performed in different plants that were under each illumination variant and at each cultivation duration period.

The method that was used provided controlled parameters in the measuring cuvette: of the CO_2_ concentration was 360 ppm, of the H_2_O concentration was 20,000 ppm, and the temperature was 23 °C. Weak pulses of blue light (460 nm) were used as the measuring light; pulses of red light (630 nm, 300 ms, 10,000 µmol m^−2^s^−1^) were used as the saturation light. Either red light (RL) or blue light (BL) was used as the actinic light; different intensities of RL and BL were used.

Dual-PAM-100 was used to measure the photosynthetic light reactions. The first saturation pulse was generated after 15 min of dark adaptation. Further saturation pulses that were periodically generated (every 20 s) were used to calculate the standard parameters of the photosynthetic light reactions. The maximal quantum yield of photosystem II (Fv/Fm), the effective quantum yields of PSI (Φ_PSI_) and PSII (Φ_PSII_), and the NPQ were automatically calculated by the Dual-PAM-100 software based on the chlorophyll fluorescence and light absorption parameters at 830 and 870 nm, in accordance to widely used equations [20,68,69,70].

Equations (1) and (2) were used to calculate the LEF and CEF in accordance with [71,72,73]:

(1) $$\mathrm{LEF}=\frac{\mathrm{SR}}{1+\mathrm{SR}}\times \mathrm{PAR}\times \mathrm{dII}\times \Phi_{\mathrm{PSII}}$$

(2) $$\mathrm{CEF}=\frac{\mathrm{SR}}{1+\mathrm{SR}}\times \mathrm{PAR}\times \left[\left(1-\mathrm{dII}\right)\times \Phi_{\mathrm{PSI}}-\mathrm{dII}\times \Phi_{\mathrm{PSII}}\right]$$

where PAR is the intensity of the actinic light (RL or BL), $\frac{\mathrm{SR}}{1+\mathrm{SR}}$ is the fraction of the actinic light absorbed by the leaves (measurement of SR was described in Section 2.2), dII is the fraction of the absorbed light distributed to photosystem II, and (1-dII) is the fraction of the absorbed light distributed to photosystem I. In accordance with an earlier proposed method [67,68,69], the dII was calculated as $\frac{\Phi_{\mathrm{PSI}}}{\Phi_{\mathrm{PSI}}+\Phi_{\mathrm{PSII}}}$, where both Φ_PSI_ and Φ_PSII_ were measured under the low intensity of the actinic light.

CO_2_ assimilation (A) and transpiration (E) were measured on the basis of GFS-3000. The photosynthetic assimilation of CO_2_ (A_hv_) was calculated as the difference between A under actinic light and A under dark conditions (after the termination of illumination). The dark respiration rate (R) was calculated as –A under dark conditions.

Fv/Fm, R, and E were measured under dark conditions before illumination was initiated by the actinic light. The dependences of A_hv_, NPQ, LEF, and CEF on the intensity of the RL and BL were also investigated in the present work. RL intensities of 0, 65, 172, 415, and 978 µmol m^−2^s^−1^ and 0, 108, 239, 425 and BL intensities of 758 µmol m^−2^s^−1^ were used. The duration of action of each actinic light intensity was 60 s; the photosynthetic parameters were measured after 50 s of illumination.

### 2.4. Measurements of Growth Parameters

Two parameter estimation methods were used in the present work to determine lettuce growth under the red and blue illumination variants during cultivation. First, all of the pallets with lettuce plants were periodically photographed (vertical position, same distance between pallet and camera, and black background were used). The standard functions (including “Threshold Color” and “Measure”) of the ImageJ 1.46 r software (the free program for analysis of images) were used to estimate the total green area in each image (Figure S1). After that, the total green area was normalized based on the quantity of plants in the pallet. However, this method could only be used as coarse estimator of the increases in the plant biomass and in the leaf area; plant growth can decrease efficiency of this method.

Second, we measured the fresh and dry weight of the lettuce leaf rosettes after 25 days of cultivation. The dry weight was measured after 6 h of drying at 100 °C. The fresh and dry weight were calculated per plant. Using these parameters were more accurate for the estimation of plant growth.

### 2.5. Statistics

Different lettuce plants were used in different experiments. Mean values, standard errors, and correlation coefficients are shown in the figures. The significant difference was estimated using Student’s *t*-test.

## 3. Results

### 3.1. Influence of Red and Blue Variants of Illumination on Photosynthetic Assimilation of CO_2_, Respiration, and Transpiration in Leaves of Lettuce Plants

**Figure 2:**
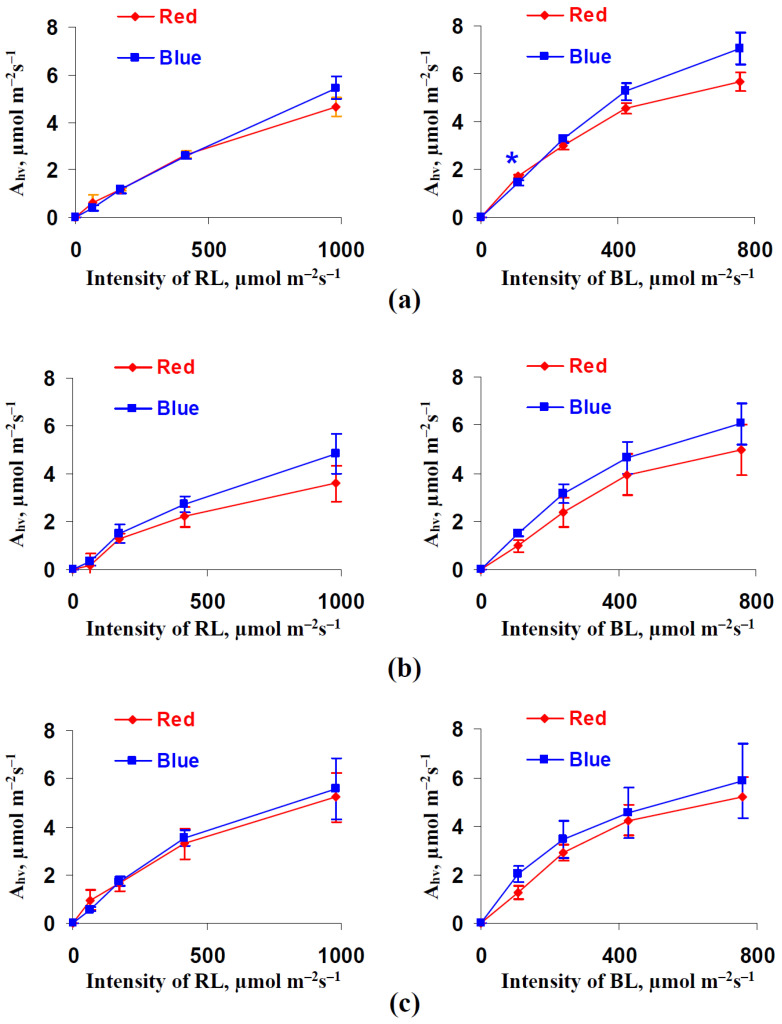
Dependences of the photosynthetic CO_2_ assimilation (A_hv_) on the intensity of the red actinic light (RL) (left panels) and blue actinic light (BL) (right panels) after 18 (**a**), 25 (**b**), and 32 (**c**) days of lettuce cultivation in the red (marked as “Red”) and blue (marked as “Blue”) illumination variants (see Figure 1) (*n* = 5–7). A_hv_ was calculated as the difference between the rate of the CO_2_ assimilation under illumination by the RL or BL and this rate under dark conditions. * differences between plants cultivated in the red and blue illumination variants were significant (*p* < 0.05).

The dependences of the photosynthetic assimilation of CO_2_ on the intensities of the red and blue actinic light were investigated during the first stage of our work. It was shown (Figure 2) that A_hv_ increased as the intensity of both RL and BL increased; the saturation tendencies were observed under the high actinic light intensities. However, the illumination variant that was used during lettuce cultivation weakly influenced the light dependences of A_hv_. There was only single point with significant differences between photosynthetic assimilations in plants that had been cultivated under red and blue variants of illumination: the red variant weakly stimulated A_hv_ at the 108 µmol m^−2^s^−1^ intensity of BL after 18 days of cultivation.

**Figure 3:**
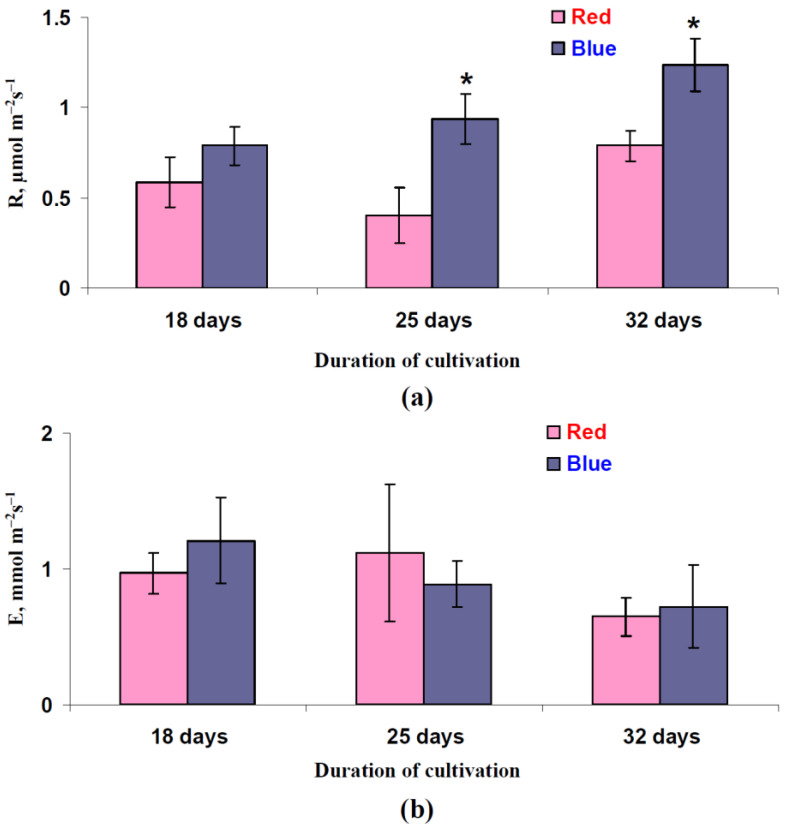
Influence of the red (marked as “Red”) and blue (marked as “Blue”) illumination variants during lettuce cultivation on rates of the dark respiration (R) (**a**) and transpiration (E) (**b**) (*n* = 5–7). Illumination spectra are shown in Figure 1. * differences between plants cultivated under the red and blue illumination variants were significant (*p* < 0.05).

In contrast, the dark respiration rate was increased in leaves of lettuce that had been cultivated under the blue illumination variant (Figure 3a). This effect was significant after 25 and 32 days of cultivation; the only tendency was observed after 18 days of this cultivation period. Importantly, the relative magnitudes of this R increased under the blue illumination variants: about 35% after 18 days, about 130% after 25 days, and about 60% after 32 days of lettuce cultivation.

The influence of variant of illumination during lettuce cultivation on transpiration was not shown in our work (Figure 3b); the last result showed that the illumination variant did not influence stomata opening in the lettuce plants.

### 3.2. Influence of Red and Blue Variants of Illumination on Parameters of Photosynthetic Light Reactions in Leaves of Lettuce Plants

Furthermore, the photosynthetic light reaction parameter dependences (Fv/Fm, NPQ, LEF, and CEF) on the intensities of the red and blue actinic light in plants that had been cultivated in the red and blue illumination variants were investigated.

**Figure 4:**
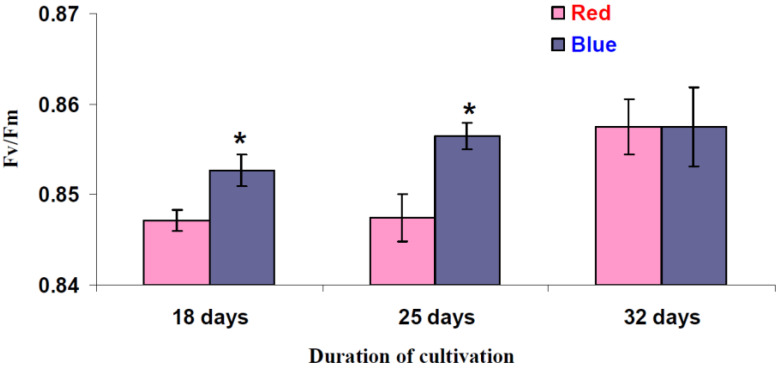
Influence of the red (marked as “Red”) and blue (marked as “Blue”) illumination variants on lettuce cultivation on the maximal quantum yield of photosystem II (Fv/Fm) (*n* = 5–7). Illumination spectra were shown in Figure 1. * differences between plants cultivated under the red and blue illumination variants were significant (*p* < 0.05).

It was shown (Figure 4) that the blue illumination variant resulted in a significant increase in the maximal quantum yield of photosystem II in the leaves of the lettuce plants after 18 and 25 days of cultivation; this effect was absent after 32 days of cultivation. However, it should be noted that this response had a very low magnitude because the relative differences between the Fv/Fm in plants grown under the red and blue illumination variants were less than 1%.

**Figure 5:**
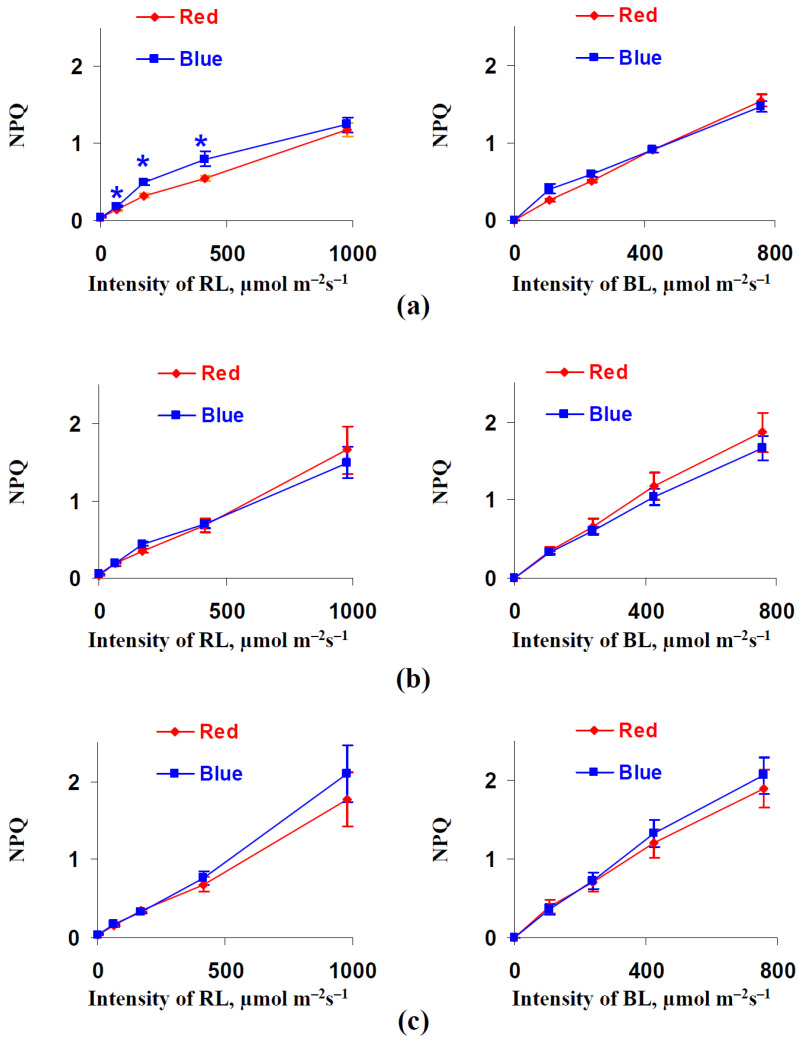
Dependences of the non-photochemical chlorophyll fluorescence quenching (NPQ) on the red actinic light (RL) intensity (left panels) and blue actinic light (BL) (right panels) after 18 (**a**), 25 (**b**), and 32 (**c**) days of lettuce cultivation under the red (marked as “Red”) and blue (marked as “Blue”) illumination variants (see Figure 1) (*n* = 5–7). * differences between plants cultivated under the red and blue illumination variants were significant (*p* < 0.05).

Figure 5 shows the influence of the illumination variants during lettuce cultivation on NPQ. This influence was absent in the investigated cases, excluding the significant increase in the NPQ that was found under the 131, 344, and 830 µmol m^−2^s^−1^ RL intensities after 18 days of lettuce cultivation under the blue illumination variant.

**Figure 6:**
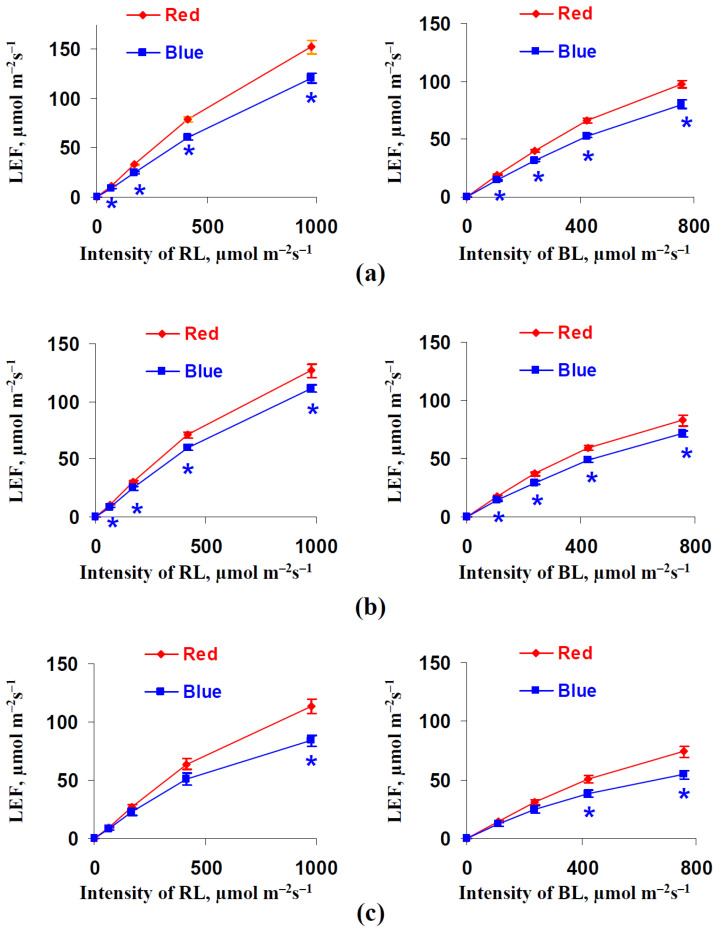
Dependences of the linear electron flow (LEF) on the intensity of the red actinic light (RL) (left panels) and blue actinic light (BL) (right panels) after 18 (**a**), 25 (**b**), and 32 (**c**) days of lettuce cultivation under the red (marked as “Red”) and blue (marked as “Blue”) illumination variants (see Figure 1) (*n* = 5–7). * differences between plants cultivated under the red and blue variants of illumination were significant (*p* < 0.05).

Figure 6 shows the influence of illumination variants during lettuce cultivation on the linear electron flow. The LEF in the leaves of plants that had been cultivated under the blue illumination variant was lower than the LEF in the leaves of plants that had been cultivated under the red illumination variant. This effect was significant in the most of investigated points (at different cultivation duration periods and at different RL or BL intensities) excluding cases with low and moderate intensities of the red and blue actinic light after 32 days of lettuce cultivation. The relative magnitudes of the LEF decrease under the blue variant were about 13–26%.

**Figure 7:**
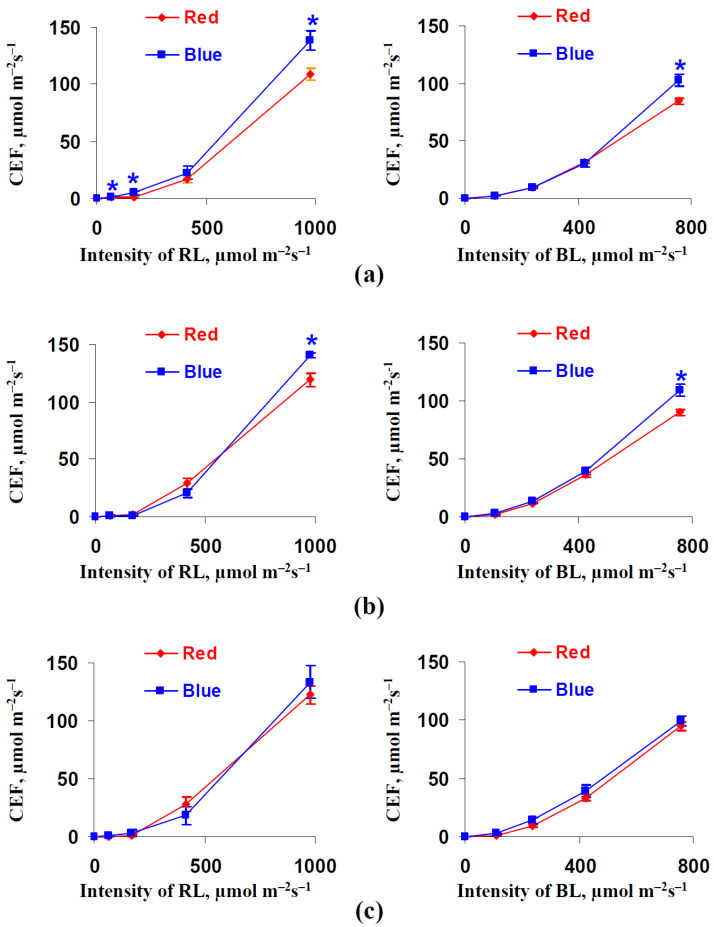
Dependences of the cyclic electron flow around photosystem I (CEF) on the intensity of the red actinic light (RL) (left panels) and blue actinic light (BL) (right panels) after 18 (**a**), 25 (**b**), and 32 (**c**) days of lettuce cultivation under the red (marked as “Red”) and blue (marked as “Blue”) illumination variants (see Figure 1) (*n* = 5–7). * differences between plants cultivated under the red and blue illumination variants were significant (*p* < 0.05).

Figure 7 shows the influence of illumination variants during lettuce cultivation on the cyclic electron flow around photosystem I. It was shown that the CEF was higher in plants after 18 and 25 days of cultivation under the blue illumination variant; this effect was absent after 32 days of lettuce cultivation. It should be noted that this significant increase in CEF was mainly observed under the maximal intensities of RL and BL; the magnitude of these changes was about 18–26%.

Thus, the results of this section show that of the illumination variant that was used during lettuce cultivation mostly influenced the linear and cyclic electron flows; the magnitudes of these effects could be tens of percentages.

### 3.3. Influence of Red and Blue Variants of Illumination on Reflectance Indices in Leaves of Lettuce Plants

The influence of the red and blue illumination variants during lettuce cultivation on the main reflectance indices in the leaves was analyzed during the next stage of the investigation. The results of this analysis are shown in Table 1.

It was shown that 20 reflectance indices from 25 investigated ones could be differed in plants that had been cultivated under the red and blue illumination variants. In particular, several reflectance indices were significantly affected by the different illumination variants at all of the investigated lettuce cultivation duration periods (18, 25, and 32 days). These indices included MCARI and TCARI (calculated on basis of reflectance at 550, 670, and 700 nm), ZMI (calculated on basis of reflectance at 710 and 750 nm), Ctr1 (calculated on basis of reflectance at 420 and 695 nm), and GM1 (calculated on the basis of reflectance at 750 and 550 nm).

It is known that these indices were mainly related to the chlorophyll content in plants, e.g., TCARI and MCARI were negatively correlated with the total concentration of chlorophylls a and b, and ZMI was positively correlated with this concentration [57,74]. Our results showed that TCARI and MCARI decreased and that ZMI increased in the plants that had been cultivated under the blue illumination variant. Considering this result, the plants that had been cultivated under the blue illumination variation was likely to have more chlorophylls than the ones that had been cultivated under the red variant.

It can be proposed that the differences that were found in the photosynthetic parameters in plants that had been cultivated under the red and blue illumination variants were related to changes in reflectance indices. Table 2 shows the correlation coefficients between the investigated reflected indices and some photosynthetic parameters, including Fv/Fm, LEF under the 978 µmol m^−2^s^−1^ intensity of the red actinic light (LEF(RL)_max_), LEF under the 758 µmol m^−2^s^−1^ intensity of the blue actinic light (LEF(BL)_max_), CEF under the 978 µmol m^−2^s^−1^ intensity of the red actinic light (CEF(RL)_max_), and the 758 µmol m^−2^s^−1^ intensity of the blue actinic light (CEF(BL)_max_). We did not analyze A_hv_ and NPQ because the differences in these parameters between plants that had been cultivated under the red and blue illumination variants were weak. Only the averaged values of the parameters were used in this analysis.

Table 2 shows that only a few of the reflectance indices were significantly correlated with the LEFs and Fv/Fm; the changes that were observed in the CEF were not significantly correlated with any of the analyzed reflectance indices. It should be noted separately that MCARI, TCARI, ZMI, and GM1 (see above) were significantly correlated with linear electron flows; MCARI, TCARI, and Ctr1 were significantly correlated with Fv/Fm.

### 3.4. Influence of Red and Blue Variants of Illumination on Quantum Yields of Photosystems I and II and dII in Leaves of Lettuce Plants

The results of the analysis of the reflectance indices (Section 3.3) showed that cultivation under the blue illumination variant increased the chlorophyll content in the leaves of the lettuce plants. In theory, the blue illumination variant should also increase both the LEF and CEF in the plants that were grown under that condition (through increasing the light absorption); however, the LEF was decreased in this illumination variant (Figure 6), and CEF was increased (Figure 7). Considering Equations (1) and (2), this decrease in the LEF and increase in the CEF could be related to a decrease in Φ_PSII_ and an increase in Φ_PSI_, respectively. Alternatively, both responses could be caused by a decrease in dII and hence increase in (1-dII). As a result, we analyzed the Φ_PSII_ and Φ_PSI_ at the maximal RL and BL intensities; dII was also investigated.

**Figure 8:**
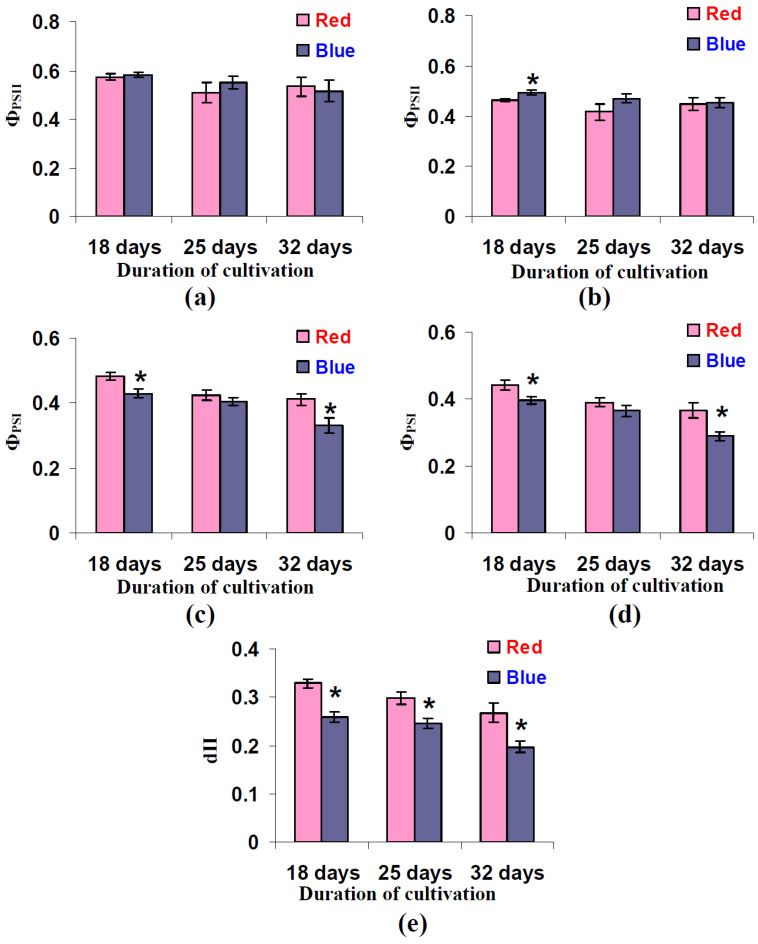
Influence of the red (marked as “Red”) and blue (marked as “Blue”) illumination variant during lettuce cultivation on the quantum yield of photosystem II (Φ_PSII_) at the 978 µmol m^−2^s^−1^ intensity of RL (**a**), Φ_PSII_ at the 758 µmol m^−2^s^−1^ intensity of BL (**b**), the quantum yield of photosystem I (Φ_PSI_) at the 978 µmol m^−2^s^−1^ intensity of RL (**c**), Φ_PSI_ at the 758 µmol m^−2^s^−1^ intensity of BL (**d**), and the fraction of the absorbed light distributed to photosystem II (dII) (**e**) (*n* = 5–7). Illumination spectra are shown in Figure 1. * differences between plants cultivated at the red and blue variants of illumination are significant (*p* < 0.05).

It was shown (Figure 8a,b) that cultivation under the blue variant of illumination weakly influenced Φ_PSII_. The increase in this parameter was significant under RL after 18 days of lettuce cultivation; a tendency towards this increase was observed in several other experimental points. In contrast, Φ_PSI_ decreased in plants after cultivation under the blue illumination variant (Figure 8c,d). This effect was significant under both RL and BL after 18 and 32 days of lettuce cultivation. An analysis of the dII (Figure 8e) showed that this parameter was significantly decreased in all of the instances where the lettuce plants had been cultivated under the blue illumination variant.

### 3.5. Influence of Red and Blue Variants of Illumination on Parameters of Growth of Lettuce

**Figure 9:**
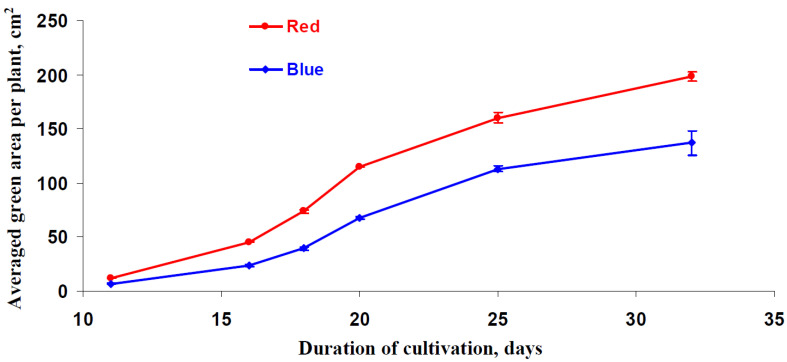
The dependence of the averaged green area per plant on duration of lettuce cultivation under the red (marked as “Red”) and blue (marked as “Blue”) illumination variants (see Figure 1).

Finally, the potential influence of the red and blue illumination variants on the lettuce growth parameters was investigated. First, the changes in the number of green areas per plant during lettuce cultivation were investigated (Figure 9); these areas were considered to be an indicator of leaf area. Color photos of the pallets (15 pots with lettuce per the pallet) were used to measure the total green area using ImageJ. After that, the averaged green area per plant was calculated for each pallet.

It was shown that the averaged green area per plant was increased as the length of the cultivation period increased, and this was the case for plants that had been grown under both illumination variants. The velocity at which this area increased decreased as the cultivation period became longer. It is likely that this effect was related to an increase in the number of leaves that were overlapping with each other. The green area per plant under the red illumination variant was more than this area that were present in the plants that had been grown under the blue variant. This effect was observed at all of the investigated lettuce cultivation duration periods.

**Figure 10:**
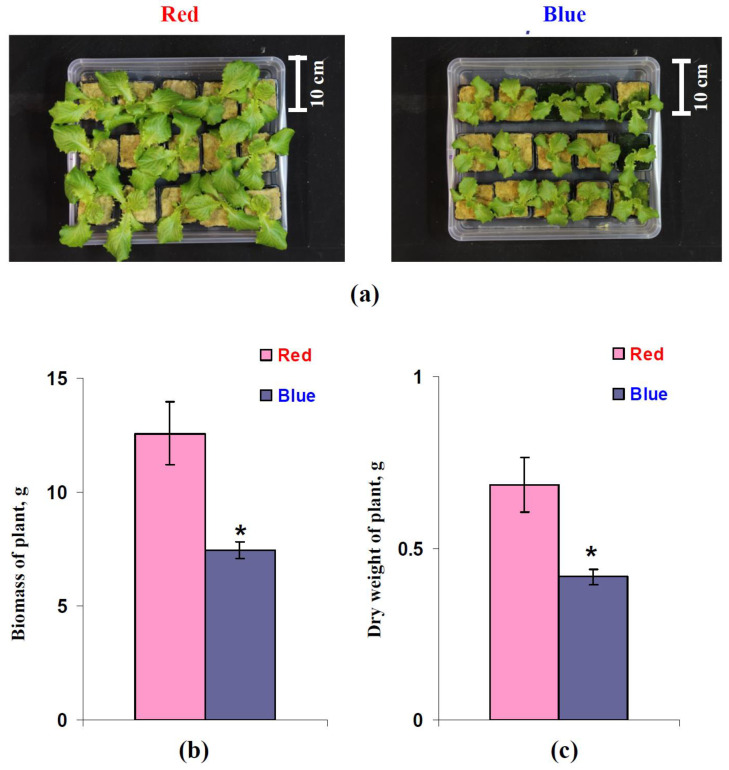
Examples of lettuce plants after 25 days of cultivation under the red (marked as “Red”) and blue (marked as “Blue”) illumination variants (**a**), the averaged biomass (**b**), and dry weight (**c**) of these plants (*n* = 8). The fresh and dry weight were calculated per plant. * differences between plants cultivated under the red and blue illumination variants were significant (*p* < 0.05).

The growth parameters of the lettuce plants, which were analyzed after 25 days of cultivation, were investigated in more detail. It was observed (Figure 10a) that the visual sizes of the lettuce plants that had been cultivated under the red illumination variant were larger than the sizes of the plants that had been grown under the blue illumination variant. Figure 10b,c show the fresh weight (total biomass) and dry weight of leaf rosettes for each lettuce plant after 25 days of cultivation. It can be seen that both parameters were decreased in the plants that had been cultivated under the blue illumination variant. The relative decreases in the total biomass and dry weight were about 41% and 39%, respectively.

## 4. Discussion

Light sources that are derived from energy-saving LEDs are a prospective tool for plant cultivation [2,3,18,49,50] because LED light can have specific narrow spectral bands; its intensity and time regime can be regulated within wide range. The influence of the light characteristics on the plants can be related to both the amount of light that is absorbed by the photosynthetic light harvesting complexes (and the further electron transfer) [1,2,3] and to the light-induced activation of the photoreceptors (e.g., phytochromes, cryptochromes, or phototropins [23,24,25]). The influence of light on plants is related to various activities, including participation as the energy source for photosynthesis [4,5,6,7], induction of numerous regulatory mechanisms [1,2,3,8,9,10,11,12,13], and stimulation of photodamage [10,14,15,16,17].

How light influences physiological plant process, including photosynthesis, is a complex problem, which is the reason why numerous studies using a diversity of plant studies have focused on this problem. Lettuce is an important agricultural plant; artificial illumination is widely used during its cultivation [49]. As a result, investigating the influence of illumination spectra on the physiological processes in lettuce during cultivation is an important task. In recent years, numerous studies (e.g., [50,51,52,75,76]) that have investigated the influence of LED illumination with different intensity ratios of red and blue light on lettuce growth, productivity, biochemical composition, and many other parameters, have been published. In particular, there are works that directly analyze the photosynthetic parameters [41,46,75,77] and leaf optical properties [44] in lettuce plants that have been cultivated under different light spectra.

However, some questions require further investigation. (i) Previous studies have shown [41,50,51,52,78] that increasing the portion of blue light during lettuce cultivation decreases the dry weight and productivity per unit of the light intensity in plants. In contrast, this increase in blue light can stimulate A_hv_ [41,47]; this effect could be related the increased stomata opening and the increased chlorophyll content. This means that there is contradiction between the changes that occur in the productivity and in the A_hv_ (which is basis of this productivity). (ii) Data about the of influence of the portion of the blue light illumination on chlorophyll content are contradictory. Some works [41,52] have shown that increasing this portion increases the chlorophyll content (mainly, total chlorophylls and chlorophyll a); other works [50] do not show any significant changes in the chlorophyll content. (iii) There are works shown the influence of the illumination spectra during lettuce cultivation on LEF [75,77]; however, investigations into the influence of these spectra on CEF are practically absent. Considering the participation of CEF in photosynthetic stress changes [8,33], the analysis of the influence of the illumination spectra during lettuce cultivation on CEF could be important.

The results of the current work show several important points. First, using a red illumination variant during cultivation increases lettuce growth (Figure 9 and Figure 10) and also increases the dry weight, indicating an increase in the plant productivity. This result is in a good accordance with the number of works that also show the stimulation of growth and productivity in lettuce being treated with red light and the decrease in these processes when the plants are being treated with blue light [41,50,51,52,78]. However, we were unable to a reveal an increase in the photosynthetic CO_2_ assimilation when using the red illumination variant (Figure 2). This result, which is in accordance with the literature data showing A_hv_ stimulation when the blue light portion increases and when the red light portion decreases [41,47] or the absence of changes in this parameter [46], means that the stimulation of A_hv_ is not likely to participate in the increase in lettuce productivity. Considering productivity as a function of the difference between the rates of photosynthesis and respiration, this increased plant productivity under the red illumination variant could be related to the low respiration rate. Our results support this proposition because the dark respiration rate under the red illumination variant is significantly lower than this rate under the blue variant (Figure 3a).

This revealed effect is supported by the results of other work [38] showing that increasing the portion of blue light and decreasing the portion of the red light during cucumber cultivation stimulates R. It should be noted that the activation of dark respiration during cultivation under blue light conditions is in a good accordance with results of work [41], which shows that increasing the portion of the blue light during lettuce cultivation induces both the decrease in the plant productivity (decreasing the dry weight) and the increase in the photosynthetic assimilation of CO_2_. The decreased R at the red illumination variant could be related to the red-light induced activation of the phytochromes because this activation can suppress the enzyme activity in the tricarboxylic acid cycle and during mitochondrial electron transport (e.g., succinate dehydrogenase, subunits of the pyruvate dehydrogenase complex, cytochrome oxidase and fumarase) [37].

It is probable that this contradiction between the absence of changes in CO_2_ assimilation and decreased production of biomass under blue light can be also related to induction of forming small sun-type leaves [38] and decreasing light interception. Considering the decrease of the area of green leaves in lettuce cultivated under the blue variant of the illumination (Figure 9), this mechanism (the decrease of leaf sizes) can also participate in the revealed increase in productivity. However, a combination of results from Figure 9 and Figure 10c shows that the dry weight per area of green leaves is 0.00429 ± 0.00015 g cm^−2^ (the increased red light) and 0.00369 ± 0.00014 g cm^−2^ (the increased blue light); i.e., elimination of difference between sizes of leaves does not eliminate the significant difference in the plant productivity.

Second, our results show that the blue illumination variant induces changes in the reflectance indices (Table 1), which are related to chlorophyll concentrations (e.g., decreases in TCARI and MCARI, which are negatively correlated with the total concentrations of chlorophylls a and b and with increases in ZMI, which is positively correlated with this concentration [57,74]). This result shows that the increase in the chlorophyll concentration that was observed in the lettuce plants in the present study is in good accordance with the literature data regarding the stimulation photosynthetic pigment synthesis under blue light conditions in plants of different spices [38,41,42,46,52].

We might expect that the increase in the chlorophyll concentration should increase both LEF and CEF, which are dependent on light absorption by the photosynthetic pigments [33,34,35]. However, the LEF in the lettuce leaves decreased under blue light illumination conditions (Figure 6); in contrast, CEF is increased under this variant (Figure 7). This means that changes in the LEF and perhaps in the CEF are related to other mechanisms. We hypothesize that this mechanism is the result of a decrease in the fraction of the absorbed light that is distributed to photosystem II (dII) in plants that were cultivated under the blue light illumination variant. This hypothesis is supported by decreased dII in these plants (Figure 8e) and by weak changes in the quantum yield of PSII (Figure 8a,b). Moreover, decreasing Φ_PSI_ under high intensities of the actinic light in plants that were cultivated under the blue light illumination variant (Figure 8c,d) corresponds to this hypothesis because the increased (1-dII) can decrease the quantum yield of PSI through stimulation of the light energy flow into photosystem I.

Revealed changes in dII could be caused by an increase in size of the light harvesting complex of photosystem I because this increase should stimulate the flow of the light energy to photosystem I. It is known [79] that the core and light harvesting complex of photosystem I can absorb light at wavelengths that are to about 710–720 nm; in contrast, the core of photosystem II and light-harvesting complex II cannot absorb light in this spectral range. ZMI, which is calculated on the basis of reflectance at 710 and 750 nm [57], can be used to reveal changes in these cores and in the light-harvesting complex of photosystem I because an increase in the sizes of these structures would decrease the reflectance at 710 nm and would thereby increase ZMI. Our results show a significant increase in this reflectance index (Table 1) that supports our hypothesis; moreover, ZMI is significantly correlated to the linear electron flow (Table 2). It is known [80] that the plants that have been cultivated under blue light can stimulate the expression of the photosynthetic complex and enzyme genes that participate in chlorophyll synthesis; this mechanism could potentially participate in the redistribution of the flow of light energy to photosystem I (the dII decrease). It should be noted that our result is in accordance with work [81] which shows that the cultivation under the blue light decreases size of the light harvesting complex of PSII because this effect can decrease dII and increase (1-dII).

**Figure 11:**
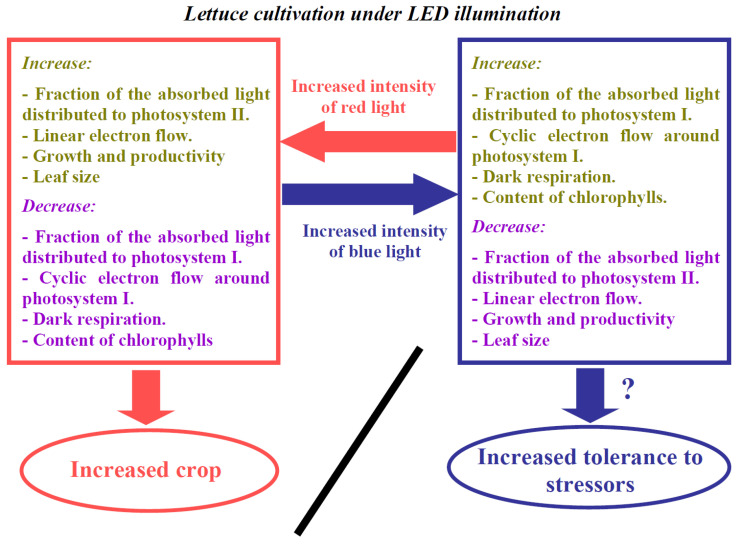
Proposed schema of changes in physiological and growth processes in lettuce during cultivation under the increased red light and blue light intensities.

Finally, the changes that were induced by cultivation under the blue light variant (the activation of CEF, respiration, and, in some cases, NPQ, the decrease in LEF, growth, and productivity) seem to be typical adaptive responses to stressors [1,3,8,10,12,16,17,33,34]. This means that our results are in accordance with hypotheses regarding the participation of blue light during plant acclimation in excess illumination conditions [82]. Considering the non-specific characteristics of the revealed changes [33,34], we suppose that increasing the portion of the blue light during lettuce cultivation can stimulate the tolerance of these plants to various stressors (Figure 11); however, this hypothesis requires future investigation. In contrast, changes that have been induced by cultivation under the red illumination variant (the increase in LEF, growth, and productivity, the decrease in CEF, respiration, and, in some cases, NPQ) should contribute to increasing the yield of the lettuce crop; however, these changes are likely to decrease the tolerance of the plant to stressors.

Thus, our analysis shows some of the characteristics of physiological processes and the growth of lettuce cultivated under illumination with the increased intensity of blue or red light. There are two main points that should be noted in particular: (i) The increase in the lettuce productivity during cultivation under the increased red light intensity can be explained by the decrease in the dark respiration. (ii) The distribution of the absorbed light energy between photosystem I and II can be differed in lettuce plants that have been cultivated under the increased intensities of red and blue light. These changes in the distribution are likely to modify rates of the linear electron transport and cyclic electron transport around photosystem I.

## 5. Conclusions

We investigated influence of the LED illumination with the increased intensities of red or the blue light during lettuce cultivation. It was shown that when the intensity of the red light increased, plants had high linear electron flow, increased productivity, decreased cyclic electron flow around photosystem I, low dark respiration, and decreased chlorophyll content estimated on basis of the reflectance indices. The opposite effects were observed when the intensity of the blue light increased. It was also shown that changes in the linear and cyclic electron flows were related to changes in the distribution of the absorbed light energy between photosystems I and II.

Finally, it should be noted that the comparison of the LED illuminations with the increased blue and red light was the main task of our work. As a result, we did not use the additional control light in our work (e.g., the white fluorescent light or white LEDs); potentially, comparison of treatment by LED illumination with the increased blue or red light to the control white light can be an interesting future task.

## Figures and Tables

**Table 1:** Influence of the red and blue illumination variants during lettuce cultivation on the reflectance indices in leaves after 18, 25, and 32 days of cultivation.

Index		18 Days		25 Days		32 Days	
		Red	Blue	Red	Blue	Red	Blue
Normalized Difference Vegetation Index (NDVI)	M	0.647	0.654	0.646	0.692	0.648	0.695
	SE	0.007	0.006	0.011	0.007	0.010	0.008
Simple Ratio Index (SR)	M	4.732	4.832	4.814	5.606	4.808	5.674
	SE	0.114	0.103	0.196	0.155	0.155	0.176
Optimized Soil-Adjusted Vegetation Index (OSAVI)	M	0.720	0.712	0.700	0.707	0.691	0.693
	SE	0.004	0.004	0.010	0.008	0.009	0.007
Simple Ratio 554/677 Greenness Index (G)	M	3.696	3.364	3.726	3.327	3.226	3.113
	SE	0.051	0.050	0.059	0.049	0.075	0.069
Modified Chlorophyll Absorption in Reflectance Index (MCARI)	M	0.542	0.455	0.488	0.354	0.394	0.292
	SE	0.019	0.016	0.024	0.015	0.020	0.015
Modified Chlorophyll Absorption in Reflectance Index 1 (MCARI1)	M	0.967	0.941	0.867	0.830	0.871	0.775
	SE	0.016	0.010	0.028	0.030	0.027	0.026
Transformed Chlorophyll Absorption Ratio Index (TCARI)	M	−0.476	−0.394	−0.430	−0.308	−0.344	−0.256
	SE	0.017	0.015	0.021	0.013	0.017	0.013
Triangular Vegetation Index (TVI)	M	35.674	34.899	32.037	31.103	32.369	29.184
	SE	0.590	0.376	1.048	1.120	1.000	0.995
Zarco-Tejada and Miller Index (ZMI)	M	1.559	1.604	1.571	1.725	1.617	1.780
	SE	0.016	0.015	0.026	0.019	0.023	0.023
Simple Ratio Pigment Index (SRPI)	M	1.158	1.168	1.118	1.139	1.129	1.154
	SE	0.012	0.012	0.014	0.016	0.013	0.027
Normalized Phaeophytinization Index (NPQI)	M	0.046	0.032	0.043	0.061	0.040	0.039
	SE	0.004	0.006	0.004	0.009	0.004	0.015
Photochemical Reflectance Index (PRI)	M	0.021	0.021	0.022	0.027	0.022	0.028
	SE	0.001	0.001	0.001	0.001	0.001	0.001
Normalized Pigment Chlorophyll Index (NPCI)	M	−0.072	−0.077	−0.055	−0.063	−0.060	−0.068
	SE	0.005	0.005	0.006	0.007	0.006	0.011
Carter Index 1 (Ctr1)	M	1.871	1.746	1.970	1.675	1.707	1.578
	SE	0.038	0.034	0.049	0.031	0.042	0.039
Carter Index 2 (Ctr2)	M	0.280	0.272	0.281	0.237	0.272	0.231
	SE	0.006	0.005	0.009	0.006	0.008	0.006
Lichtenthaler Index 1 (Lic1)	M	0.778	0.770	0.781	0.789	0.760	0.781
	SE	0.004	0.003	0.006	0.005	0.007	0.006
Lichtenthaler Index 2 (Lic2)	M	0.765	0.825	0.738	0.804	0.818	0.824
	SE	0.015	0.017	0.018	0.022	0.016	0.024
Structure Intensive Pigment Index (SIPI)	M	0.739	0.731	0.742	0.761	0.726	0.750
	SE	0.005	0.004	0.007	0.005	0.007	0.007
Gitelson and Merzlyak Index 1 (GM1)	M	2.241	2.343	2.270	2.610	2.352	2.709
	SE	0.036	0.035	0.058	0.046	0.052	0.054
Gitelson and Merzlyak Index 2 (GM2)	M	2.469	2.570	2.515	2.895	2.600	3.015
	SE	0.045	0.042	0.073	0.058	0.062	0.070
Anthocyanin Reflectance Index 1 (ARI1)	M	−0.428	−0.418	−0.544	−0.605	−0.493	−0.690
	SE	0.022	0.015	0.060	0.038	0.018	0.055
Anthocyanin Reflectance Index 2 (ARI2)	M	−0.231	−0.229	−0.247	−0.289	−0.251	−0.309
	SE	0.010	0.008	0.016	0.014	0.012	0.018
Carotenoid Reflectance Index 1 (CRI1)	M	4.825	4.683	5.757	6.276	5.006	6.493
	SE	0.174	0.115	0.389	0.365	0.220	0.432
Carotenoid Reflectance Index 1 (CRI2)	M	4.397	4.265	5.213	5.670	4.513	5.803
	SE	0.157	0.107	0.333	0.334	0.210	0.387
Ratio Difference Vegetation Index (RDVI)	M	0.612	0.607	0.578	0.584	0.579	0.566
	SE	0.005	0.004	0.012	0.012	0.011	0.010

The indices were described in Section 2.2 in detail; their equations were shown in Table S1. Means (M) and standard errors (SE) are shown in the table. Dark grey background shows indices that differed significantly (*p* < 0.05) in plants that had been cultivated under the red and blue illumination variants. The light grey background shows the indices that were not significantly different in plants that had been cultivated under these illumination variants. Significances were independently calculated for plants after 18, 25, and 32 days of cultivation.

**Table 2:** Correlation coefficients between reflectance indices and parameters of photosynthetic light reactions including the maximal quantum yield of photosystem II (Fv/Fm), linear electron flows under the 978 µmol m^−2^s^−1^ RL intensity (LEF(RL)_max_) and 758 µmol m^−2^s^−1^ BL intensity (LEF(BL)_max_), and cyclic electron flows around photosystem I under the 978 µmol m^−2^s^−1^ RL intensity (CEF(RL)_max_) and 758 µmol m^−2^s^−1^ BL intensity (CEF(BL)_max_).

Index	Fv/Fm	LEF(RL)_max_	LEF(BL)_max_	CEF(RL)_max_	CEF(BL)_max_
NDVI	0.656	−0.742	−0.777	0.692	0.716
SR	0.648	−0.767	−0.802	0.662	0.688
OSAVI	−0.618	0.755	0.744	−0.184	−0.177
G	−0.967	0.856	0.859	−0.677	−0.664
MCARI	−0.910	0.956	0.971	−0.702	−0.709
MCARI1	−0.664	0.893	0.910	−0.482	−0.490
TCARI	0.918	−0.955	−0.969	0.718	0.724
TVI	−0.634	0.878	0.894	−0.449	−0.457
ZMI	0.779	−0.875	−0.902	0.687	0.691
SRPI	0.034	0.045	0.044	0.239	0.134
NPQI	0.071	0.099	0.044	0.090	0.290
PRI	0.666	−0.805	−0.839	0.616	0.636
NPCI	0.028	−0.153	−0.155	−0.197	−0.103
Ctr1	−0.924	0.797	0.814	−0.683	−0.685
Ctr2	−0.734	0.804	0.837	−0.697	−0.718
Lic1	−0.178	−0.063	−0.101	0.217	0.254
Lic2	0.853	−0.636	−0.630	0.693	0.662
SIPI	0.171	−0.334	−0.379	0.399	0.470
GM1	0.763	−0.861	−0.889	0.703	0.709
GM2	0.755	−0.862	−0.891	0.680	0.689
ARI1	−0.535	0.810	0.836	−0.417	−0.424
ARI2	−0.674	0.834	0.867	−0.515	−0.541
CRI1	0.408	−0.696	−0.726	0.421	0.445
CRI2	0.386	−0.675	−0.705	0.419	0.446
RDVI	−0.559	0.811	0.817	−0.270	−0.273

The correlation coefficients were calculated on the basis of the averaged values of the analyzed parameters shown in Figure 5, Figure 6 and Figure 7 and in Table 1. Six averaged values (after 18, 25, and 32 days of cultivation under the red and blue illumination variants) were used to calculate the correlation coefficients. The dark grey background shows significant correlation coefficients (*p* < 0.05); the light grey background shows non-significant correlation coefficients.

## Data Availability

The data presented in this study are available upon request from the corresponding author.

## Supplementary Materials

The following supporting information can be downloaded at: https://www.mdpi.com/article/10.3390/biology11010060/s1. Figure S1: Example of the estimation of the green area on plants using ImageJ, including the initial image (a) and image with green areas marked (b); Table S1: List of reflectance indices automatically calculated by the PolyPen RP 410 UVIS software in accordance with [44] and used in the present work.
