# Supplementary material for: Ratio of Intensities of Blue and Red Light at Cultivation Influences Photosynthetic Light Reactions, Respiration, Growth, and Reflectance Indices in Lettuce
Source: Biology (Basel). 2022 Jan 1;11(1):60. doi: 10.3390/biology11010060 (PMC8772897; doi:10.3390/biology11010060)
Supplement: Supplementary file 1 [file biology-11-00060-s001.zip › Figure S1.pdf]

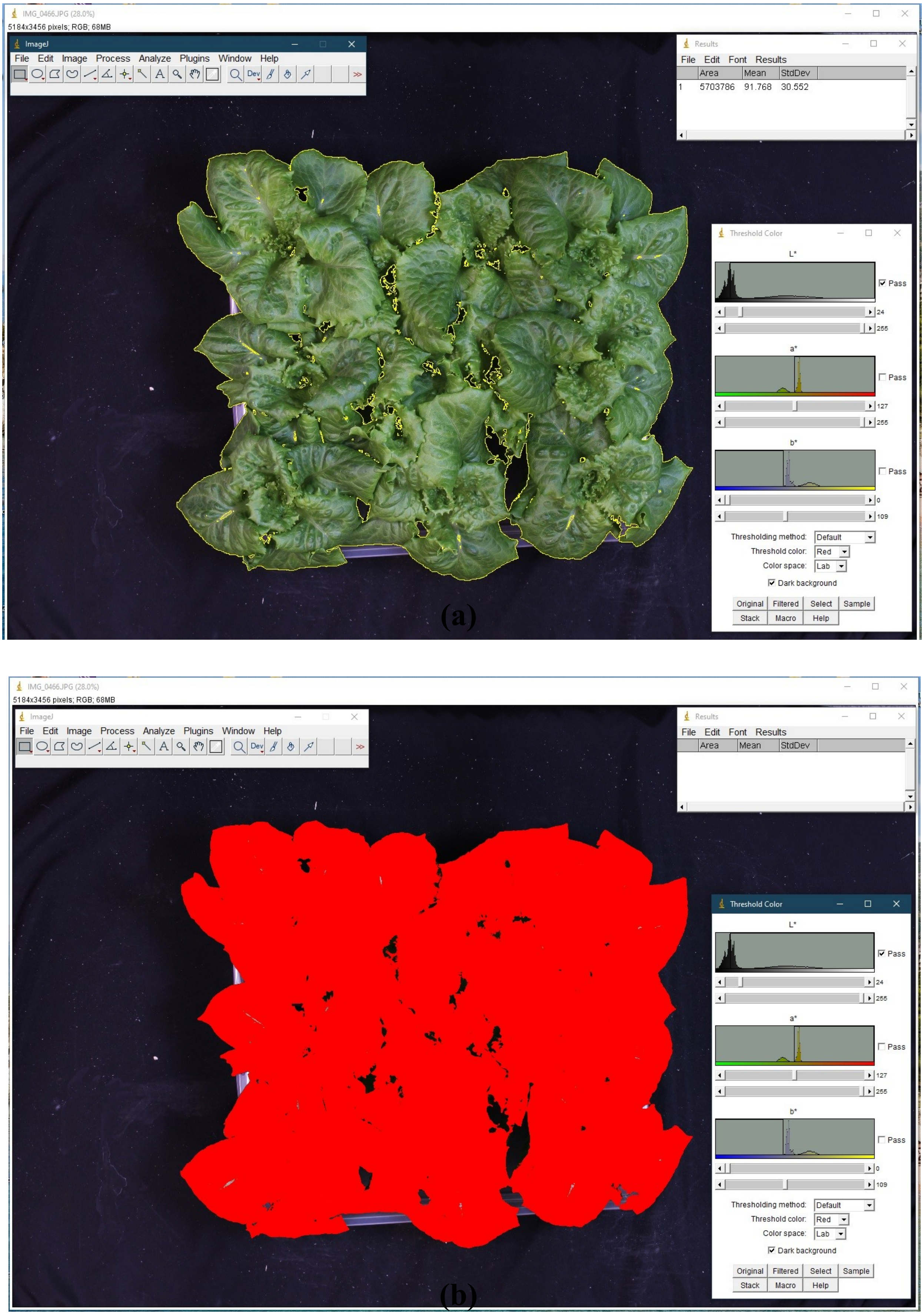

Figure S1. Example of estimation of green area of plants (for the red variant of illumination) with using ImageJ including the initial image **(a)** and image with marked green area (red color) **(b)**.
