# Supplementary material for: Ratio of Intensities of Blue and Red Light at Cultivation Influences Photosynthetic Light Reactions, Respiration, Growth, and Reflectance Indices in Lettuce
Source: Biology (Basel). 2022 Jan 1;11(1):60. doi: 10.3390/biology11010060 (PMC8772897; doi:10.3390/biology11010060)
Supplement: Supplementary file 1 [file biology-11-00060-s001.zip › Table S1.pdf]

Table S1. List of reflectance indices automatically calculated by the software of PolyPen RP 410 UVIS in accordance with [54] and used in the work.

Index	Abbreviation	Equation	Reference
Normalized Difference Vegetation Index	NDVI	$$NDVI = \frac{R_{NIR} - R_{RED}}{R_{NIR} + R_{RED}}$$ where R_{NIR} and R_{RED} are reflectance at near infrared (NIR) and red spectral regions.	[55]
Simple Ratio Index	SR	$$SR = \frac{R_{NIR}}{R_{RED}}$$ where R_{NIR} and R_{RED} are reflectance at near infrared (NIR) and red spectral regions.	[55]
Optimized Soil-Adjusted Vegetation Index	OSAVI	$$OSAVI = \frac{(1+L)(R_{790} - R_{670})}{R_{790} + R_{670} + L}$$ where R_{790} and R_{670} are reflectance at 790 and 670 nm, L = 0.16 is correction coefficient.	[56]
Simple Ratio 554/677 Greenness Index	G	$$G = \frac{R_{554}}{R_{677}}$$ where R_{677} and R_{554} are reflectance at 677 and 554 nm.	[57]
Modified Chlorophyll Absorption in Reflectance Index	MCARI	$$MCARI = [(R_{700} - R_{670}) - 0.2(R_{700} - R_{550})]\frac{R_{700}}{R_{670}}$$ where R_{700}, R_{670}, and R_{550} are reflectance at 700, 670, and 550 nm.	[57,58]
Modified Chlorophyll Absorption in Reflectance Index 1	MCARI1	$$MCARI1 = 1.2[2.5(R_{790} - R_{670}) - 1.3(R_{790} - R_{550})]$$ where R_{790}, R_{670}, and R_{550} are reflectance at 790, 670, and 550 nm.	[57,58]
Transformed Chlorophyll Absorption Ratio Index	TCARI	$$TCARI = 3\left[(R_{700} - R_{670}) - 0.2(R_{700} - R_{550})\frac{R_{700}}{R_{670}}\right]$$ where R_{700}, R_{670}, and R_{550} are reflectance at 700, 670, and 550 nm.	[58]
Triangular Vegetation Index	TVI	$$TVI = 0.5[120(R_{750} - R_{550}) - 200(R_{670} - R_{550})]$$ where R_{750}, R_{670}, and R_{550} are reflectance at 750, 670, and 550 nm.	[57,58]
Zarco-Tejada and Miller Index	ZMI	$$ZMI = \frac{R_{750}}{R_{710}}$$ where R_{750} and R_{710} are reflectance at 750 and 710 nm.	[57]
Simple Ratio Pigment Index	SRPI	$$SRPI = \frac{R_{430}}{R_{680}}$$ where R_{680} and R_{430} are reflectance at 680 and 430 nm.	[59]
Normalized Phaeophytinization Index	NPQI	$$NPQI = \frac{R_{415} - R_{435}}{R_{415} + R_{435}}$$ where R_{435} and R_{415} are reflectance at 435 and 415 nm.	[57]
Photochemical Reflectance Index	PRI	$$PRI = \frac{R_{531} - R_{570}}{R_{531} + R_{570}}$$	[60-62]

		where R_{570} and R_{531} are reflectance at 570 and 531 nm.	
Normalized Pigment Chlorophyll Index	NPCI	$$NPCI = \frac{R_{680} - R_{430}}{R_{680} + R_{430}}$$ where R_{680} and R_{430} are reflectance at 680 and 430 nm.	[63]
Carter Index 1	Ctr1	$$Ctr1 = \frac{R_{695}}{R_{420}}$$ where R_{695} and R_{420} are reflectance at 695 and 420 nm.	[57]
Carter Index 2	Ctr2	$$Ctr2 = \frac{R_{695}}{R_{760}}$$ where R_{695} and R_{760} are reflectance at 695 and 760 nm.	[57]
Lichtenthaler Index 1	Lic1	$$Lic1 = \frac{R_{790} - R_{680}}{R_{790} + R_{680}}$$ where R_{790} and R_{680} are reflectance at 790 and 680 nm.	[57]
Lichtenthaler Index 2	Lic2	$$Lic2 = \frac{R_{440}}{R_{690}}$$ where R_{690} and R_{440} are reflectance at 690 and 440 nm.	[57]
Structure Intensive Pigment Index	SIPI	$$SIPI = \frac{R_{790} - R_{450}}{R_{790} + R_{650}}$$ where R_{790}, R_{650}, and R_{450} are reflectance at 790, 650, and 450 nm.	[64]
Gitelson and Merzlyak Index 1	GM1	$$GM1 = \frac{R_{750}}{R_{550}}$$ where R_{750} and R_{550} are reflectance at 750 and 550 nm.	[65]
Gitelson and Merzlyak Index 2	GM2	$$GM2 = \frac{R_{750}}{R_{700}}$$ where R_{750} and R_{700} are reflectance at 750 and 700 nm.	[65]
Anthocyanin Reflectance Index 1	ARI1	$$ARI1 = \frac{1}{R_{550}} - \frac{1}{R_{700}}$$ where R_{700} and R_{550} are reflectance at 700 and 550 nm.	[66]
Anthocyanin Reflectance Index 2	ARI2	$$ARI2 = R_{800}\left(\frac{1}{R_{550}} - \frac{1}{R_{700}}\right)$$ where R_{800}, R_{700}, and R_{550} are reflectance at 800, 700, and 550 nm.	[66]
Carotenoid Reflectance Index 1	CRI1	$$CRI1 = \frac{1}{R_{510}} - \frac{1}{R_{550}}$$ where R_{550} and R_{510} are reflectance at 550 and 510 nm.	[67]
Carotenoid Reflectance Index 1	CRI2	$$CRI2 = \frac{1}{R_{510}} - \frac{1}{R_{700}}$$ where R_{700} and R_{510} are reflectance at 700 and 510 nm.	[67]
Ratio Difference Vegetation Index	RDVI	$$RDVI = \frac{R_{800} - R_{670}}{(R_{800} + R_{670})^{1/2}}$$ where R_{800} and R_{670} are reflectance at 800 and 670 nm.	[57]
